# Gut Microbiota-Driven Neuroimmune Crosstalk in Ulcerative Colitis: From Mechanisms to Therapeutic Implications

**DOI:** 10.3390/biomedicines14102361

**Published:** 2026-10-16

**Authors:** Lihan Yu, Lei He, Yanjun Lu

**Affiliations:** 1 Department of Laboratory Medicine, Tongji Hospital, Tongji Medical College, Huazhong University of Science and Technology, Wuhan 430074, China; lihan_yu2025@163.com; 2 Department of Blood Transfusion, Tongji Hospital, Tongji Medical College, Huazhong University of Science and Technology, Wuhan 430074, China

**Keywords:** ulcerative colitis, gut microbiota, neuroimmune crosstalk, mucosal immunity, enteric nervous system, microbial metabolites

## Abstract

Ulcerative colitis (UC) is a chronic inflammatory disease in which epithelial barrier injury, microbial perturbation, dysregulated mucosal immunity, and altered neural signaling converge. Although dysbiosis is frequently reported in UC, taxonomic shifts alone do not explain how microbial signals are translated into mucosal inflammation, urgency, pain, relapse, or heterogeneous therapeutic responses. This review develops a focused framework of gut microbiota -driven neuroimmune crosstalk, defined as bidirectional communication through which microbial communities and their metabolites regulate epithelial, neuronal, glial, enteroendocrine, and immune compartments, while neural and immune signals reshape barrier function and microbial ecology. We synthesize evidence on short-chain fatty acids, tryptophan-derived indoles, serotonin signaling, bile acids, microbe-associated molecular patterns, vagal anti-inflammatory reflexes, enteric neurons, sensory neurons, enteric glia, macrophages, dendritic cells, T cells, innate lymphoid cells, mast cells, and neutrophils. Therapeutic opportunities include diet, prebiotics, probiotics, postbiotics, fecal microbiota transplantation, metabolite-based interventions, vagus nerve stimulation, neuropeptide or serotonin pathway modulation, and combination strategies with established anti-inflammatory therapies. UC-specific evidence is emphasized where available, with explicit distinction between human UC findings, broader inflammatory bowel disease (IBD) evidence, experimental colitis models, and general mechanistic studies. A microbiota–neuroimmune framework may help move UC research beyond descriptive dysbiosis toward mechanism-guided precision therapy; however, most causal neuroimmune pathways still require validation in well-phenotyped human UC cohorts, and the proposed endotypes in this review are hypothesis-generating and require prospective validation.

## 1. Introduction

Ulcerative colitis (UC) is histopathologically characterized by relapsing mucosal inflammation, epithelial barrier disruption, crypt architectural distortion, and immune-cell infiltration in the colonic lamina propria [1]. Although current therapies targeting cytokines, leukocyte trafficking, Janus kinase signaling, or sphingosine-1-phosphate pathways have substantially improved patient outcomes [2,3], primary nonresponse, secondary loss of response, persistent symptoms, and relapse remain frequent challenges. These limitations argue against viewing UC as a linear cytokine disorder. A more useful model is that of a network disease in which epithelial, microbial, metabolic, neural, and immune compartments interact over time.

Microbiota research has been central to this conceptual shift. Broad structural and functional differences have been reported in inflammatory bowel disease (IBD) microbiomes [4,5]. In UC, reduced representation of selected obligate anaerobes and butyrate-producing species has been documented [6], whereas altered bile acid metabolism [7,8], impaired microbial tryptophan-AhR ligand production [9], and mucosal biofilms [10] provide distinct functional or spatial examples. The healthy colonic mucosa maintains host –microbial equilibrium through coordinated mucus, epithelial, immune, and repair programs, with mucus glycosylation contributing directly to this interface [11]. During active inflammation, epithelial oxygenation, nitrate availability, bleeding, altered transit, diet, and medication can reshape the microbial habitat; dysbiosis is therefore both a potential upstream contributor to and a downstream product of UC pathology. Taxonomic shifts alone do not specify which microbial functions are lost, whether microbial ligands gain abnormal access to tissue, or how these changes are translated into inflammatory and sensory phenotypes.

In the proposed model, a disease-amplifying loop can emerge when reduced microbial metabolite production and barrier injury coexist. Lower short-chain fatty acid (SCFA) availability may impair colonocyte metabolism and regulatory immune tone [6,12,13,14]. Deficient indole–aryl hydrocarbon receptor (AhR) signaling may weaken epithelial repair [9,15], whereas reduced secondary bile acid signaling may lessen epithelial and myeloid restraint [7,8,16,17,18]. Mucus or epithelial disruption can increase microbe-associated molecular pattern (MAMP) access to epithelial and myeloid compartments, promoting macrophage and dendritic cell activation and neutrophil recruitment [10,19,20,21,22]. The resulting mediators may alter enterochromaffin signaling [23,24,25,26,27,28], enteric and sensory neuronal activity [20,29,30,31,32,33,34,35,36], and enteric glial responses [37,38,39,40], thereby linking tissue inflammation to secretion, motility, urgency, and pain. Conversely, inflammation-associated changes in oxygenation, exudation, motility, diet, and drug exposure may reshape the microbial habitat [5,7]. This reciprocal relationship provides the organizing basis for the proposed framework while remaining a hypothesis that has not yet been demonstrated as a complete causal sequence in human UC.

This review therefore focuses on microbiota-driven neuroimmune crosstalk rather than on the broad gut–brain axis or a catalog of UC-associated microbes. The colonic mucosa contains epithelial and enteroendocrine cells, enteric neurons and glia, autonomic and sensory fibers, and diverse immune populations that exchange microbial metabolites, neurotransmitters, neuropeptides, cytokines, and barrier-derived signals. Experimental studies demonstrate the microbial regulation of serotonin-related pathways, stress-to-enteric nervous system (ENS) signaling, and nociceptor-microbiota feedback [23,24,26,29,30], whereas human UC studies support selected mucosal neurochemical changes and associations among residual symptoms, visceral hypersensitivity, and mast-cell activity [25,31,41]. The complete microbial–neural–immune sequence has not yet been demonstrated in human UC.

This framework is clinically relevant because inflammation, urgency, pain, relapse, stress sensitivity, and treatment heterogeneity do not always change in parallel. Immune-directed therapy confirms the central role of mucosal inflammation but may not synchronously normalize microbial metabolism, barrier repair, or neural and glial states. We therefore organize the evidence around signal transfer across the colonic mucosal ecosystem and explicitly distinguish direct human UC findings from broader inflammatory bowel disease (IBD) evidence and experimental mechanisms.

## 2. Literature Strategy and Evidence Grading

This mechanism-focused narrative review searched PubMed as the bibliographic database. The search was performed on 26 September 2026 using eight predefined queries covering UC, microbiota, microbial metabolites, neural and glial pathways, immune signaling, and candidate interventions. The searches yielded 7528 records before PubMed identifier (PMID)-based deduplication and 6748 unique records. Full field-tagged search strings, date limits, query-level yields, deduplication procedures, and eligibility criteria are provided in Supplementary File S1.

Study selection was iterative and mechanism oriented rather than a Preferred Reporting Items for Systematic Reviews and Meta-Analyses (PRISMA)-style screening process. The final synthesis included 78 references, prioritizing human UC trials, longitudinal cohorts, and mucosal tissue studies, followed by broader inflammatory bowel disease (IBD) evidence and experimental studies when UC-specific data were unavailable. Primary studies and directly relevant positive, null, and negative findings were considered; descriptive taxonomy without functional interpretation and non-UC evidence without clear mechanistic relevance were de-emphasized. Because intermediate exclusions were not prospectively recorded, no PRISMA screening counts were reconstructed.

Evidence context was classified using the following labels: A, direct human UC intervention; B, human UC observational or tissue evidence; C, human IBD evidence not specific to UC; D, in vivo experimental evidence; E, ex vivo, in vitro, or foundational non-UC mechanism; and H, author-derived hypothesis. These labels describe study context and inferential distance rather than formal risk of bias. Table 1 summarizes module-level evidence boundaries and priority questions, and Supplementary File S2 provides the evidence matrix for 30 pivotal studies.

## 3. Conceptual Framework: Microbiota-Driven Neuroimmune Crosstalk

### 3.1. Definition and Scope

Microbiota-driven neuroimmune crosstalk refers to bidirectional communication in which microbial communities and their products regulate neural, glial, epithelial, enteroendocrine, and immune compartments, while neural and immune signals in turn reshape barrier function and microbial ecology. In UC, this crosstalk is best examined primarily at the colonic mucosal interface rather than through systemic gut–brain signaling axis alone. Relevant cellular and microbial components include luminal microbes, mucus, epithelial cells, enterochromaffin cells, enteric neurons, vagal and sympathetic fibers, sensory neurons, enteric glia, macrophages, dendritic cells, T cells, innate lymphoid cells, mast cells, eosinophils, and neutrophils. A comprehensive list of key cellular and molecular components is provided in Table 2.

### 3.2. Inputs: Microbial Functions, Metabolites, and Ligands

The proposed sequence is as follows: dysbiosis alters the local pool of metabolites and microbe-associated molecular patterns (MAMPs); epithelial, enteroendocrine, neuronal, glial, and immune cells sense these signals; and neuroimmune pathways then determine whether the mucosa returns to tolerance and repair or shifts toward persistent inflammation. MAMPs constitute a distinct class of input, separate from metabolites. Their effects depend on localization and barrier access: controlled sensing contributes to defense in an intact mucosa, whereas mucus depletion, epithelial injury, or ulceration can expose epithelial and myeloid compartments, as well as potentially neural or glial sensors, to the same ligands. Spatial access may therefore matter as much as abundance.

This framework explains how microbial perturbation can generate inflammatory and sensory manifestations in parallel. It also explains why symptoms may not parallel endoscopic severity: neural sensitization, mast cell–nerve interactions, serotonin signaling, and glial activation can persist after partial inflammatory resolution.

UC is particularly well-suited to this framework because its inflammation is mucosal and colonic, and the mucosa is exposed to high microbial density and metabolite flux. Barrier defects increase microbial ligand access to immune and neural structures. Symptoms such as urgency, tenesmus, pain, altered motility, and stress sensitivity implicate neural involvement. Standard anti-inflammatory therapies do not necessarily restore microbial metabolite production, vagal tone, enteric glial homeostasis, or sensory neuron thresholds. A neuroimmune framework therefore complements, rather than replaces, immune-centered models.

### 3.3. Mucosal Transducers and Neuroimmune Effectors

Epithelial and enteroendocrine cells govern luminal compartmentalization and translate nutrients, microbial products, mechanical stimuli, and inflammation into barrier, secretory, hormonal, and immune outputs [23,59]. Immune populations integrate these cues with epithelial and stromal signals, creating the immediate tissue context in which metabolite and ligand sensing is translated into biological effect.

Enteric, sensory, and autonomic neurons, together with enteric glia, provide local and system-level regulatory routes. Their effects vary with circuit, receptor, anatomical compartment, and disease stage [30,32,37]. Vagal anti-inflammatory biology and the principal vagus nerve stimulation (VNS) pilot study in IBD illustrate the importance of separating foundational mechanisms from UC-specific clinical evidence; that pilot enrolled patients with Crohn’s disease [47]. This distinction guides the interpretation of neural and glial pathways throughout this review.

### 3.4. Feedback, Disease States, and Symptom-Inflammation Discordance

The proposed network operates as a feedback system rather than a one-way cascade. Loss of microbial fermentation may reduce epithelial fuel and regulatory immune support [6,12,13,14], while impaired indole–AhR [9,15] and bile acid receptor signaling [7,8,16,17,18] may weaken repair and myeloid restraint. When mucus and epithelial integrity fail, abnormal microbial access can promote macrophage activation and neutrophil-associated tissue injury [10,19,21,22]. Inflammatory mediators can alter enteric and sensory neuronal activity and enteric glial state, as supported mainly by experimental studies [20,29,30,39,40]. Microbiota and SCFAs regulate enterochromaffin cell serotonin experimentally [23,24], whereas UC studies report altered serotonin-related pathways [25,26,27,28]. Substance P has context-dependent effects in experimental colitis [33,34], and UC studies report altered NPY or other neuropeptide patterns [32,35,36]. Together, these component findings provide a plausible bridge between dysbiosis, mucosal inflammation, secretion, motility, urgency, and pain, but the complete sequence remains unproven in human UC.

**Figure 1:**
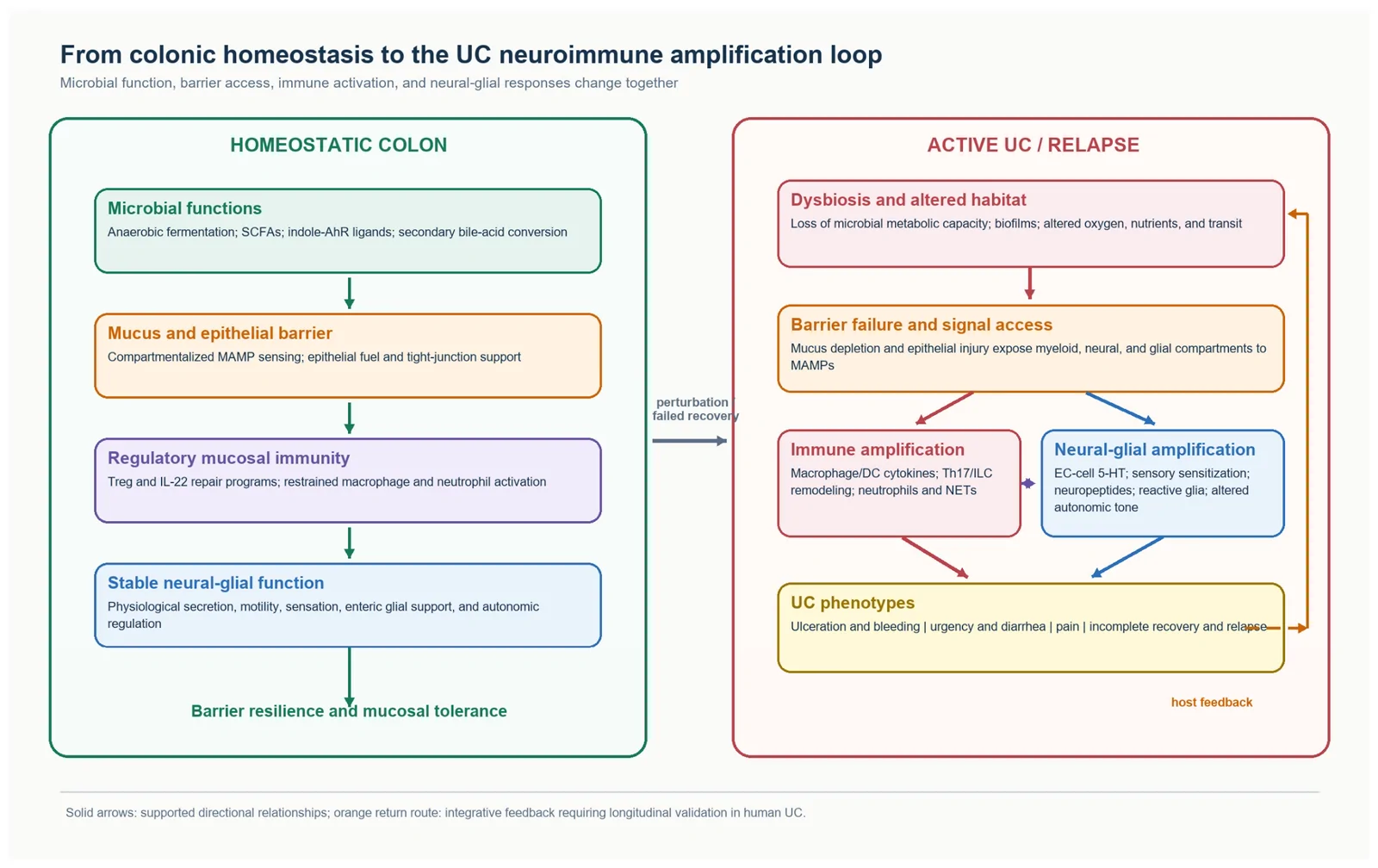
Transition from colonic homeostasis to a proposed UC neuroimmune amplification loop. In homeostasis, microbial fermentation, indole–AhR, and bile acid signaling support epithelial metabolism, barrier integrity, regulatory immunity, and stable neural–glial function. During active UC or relapse, altered microbial function and the inflammation-shaped habitat may converge with barrier failure, MAMP access, immune activation, and neural–glial responses. Solid arrows summarize component-level relationships supported across human observational and experimental studies; they do not establish the complete sequence in human UC. The orange return route is an author-derived feedback hypothesis that requires longitudinal validation. Abbreviations: AhR, aryl hydrocarbon receptor; EC, enterochromaffin; ENS, enteric nervous system; IBD, inflammatory bowel disease; ILC, innate lymphoid cell; MAMP, microbe-associated molecular pattern; NET, neutrophil extracellular trap; SCFA, short-chain fatty acid; UC, ulcerative colitis.

The direction and weight of each edge are disease-state dependent. During active UC, barrier breach and cytokine exposure may dominate the network and make a microbial change appear causal even when it is partly inflammation-driven. During recovery, mucosal healing, microbial metabolism, autonomic tone, and sensory thresholds may normalize at different rates. This may help explain why dysbiosis, barrier dysfunction, or sensory abnormalities persist or resolve asynchronously, and why residual urgency or pain can coexist with endoscopic improvement. Longitudinal sampling across flare, treatment, and remission is therefore required to distinguish driver, consequence, and compensation and to test the integrated loop shown in Figure 1.

During remission, inflammatory burden, barrier recovery, microbial function, and sensory thresholds may recover at different rates. Persistent urgency or pain can reflect residual inflammation or coexist with visceral hypersensitivity and mast-cell activity [31,41]. Objective assessment is therefore essential before symptoms are attributed to active inflammation or classified as non-inflammatory.

Selected fecal microbiota transplantation (FMT) protocols provide Level A proof-of-concept for improved short-term remission outcomes, although microbial mediation, donor effects, durability, and mechanisms remain unresolved [51,52,60,61]. The framework is intended to organize such heterogeneous evidence into testable relationships. It predicts that longitudinal, anatomically matched measurements across inflammatory, microbial, metabolic, spatial, and symptom domains will explain clinical heterogeneity better than any single data layer.

Figure 1 provides a state-based overview of the pathology-dysbiosis feedback loop.

## 4. Microbial Signals as Upstream Regulators

**Figure 2:**
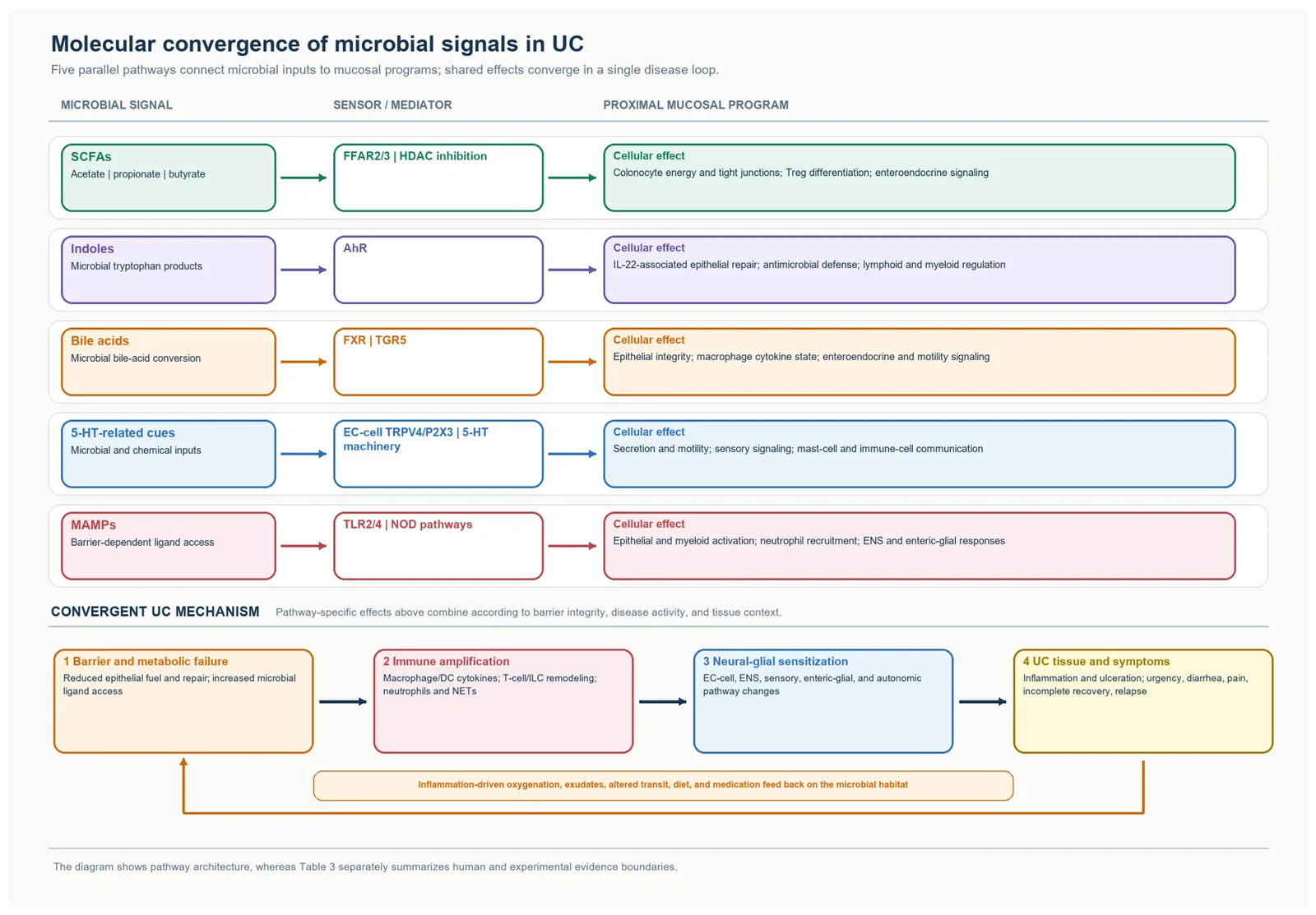
Parallel microbial signaling pathways and their convergence in UC. Five non-crossing lanes link SCFAs, indoles, bile acids, 5-HT-related cues, and MAMPs to candidate sensors or mediators and proximal mucosal programs. The figure depicts mechanistic routing and convergence; it does not grade evidence strength or imply that the complete sequence is established in human UC. Abbreviations: 5-HT, 5-hydroxytryptamine; AhR, aryl hydrocarbon receptor; DC, dendritic cell; EC, enterochromaffin; ENS, enteric nervous system; FFAR, free fatty acid receptor; FXR, farnesoid X receptor; HDAC, histone deacetylase; ILC, innate lymphoid cell; IL, interleukin; MAMP, microbe-associated molecular pattern; NET, neutrophil extracellular trap; NOD, nucleotide-binding oligomerization domain; SCFA, short-chain fatty acid; TGR5, G protein-coupled bile acid receptor 1; TLR, Toll-like receptor; Treg, regulatory T cell; UC, ulcerative colitis.

In UC, microbial metabolites are best understood as functional signal classes rather than as isolated molecules. SCFAs anchor barrier–metabolic and tolerogenic circuits; tryptophan metabolites and serotonin bridge epithelial, enterochromaffin, neural, and immune compartments; and bile acids couple microbial transformation to epithelial, myeloid, and motility pathways. Microbial ligands differ from metabolites: their effects depend on ligand identity, dose, receptor, compartment, and host state, and their pro-inflammatory impact may increase when barrier failure permits abnormal access to deeper mucosal structures. Table 3 summarizes evidence provenance and uncertainty, whereas Figure 2 depicts candidate mechanistic routing and convergence.

### 4.1. Short-Chain Fatty Acids

SCFAs—particularly acetate, propionate, and butyrate—are generated by bacterial fermentation of dietary fibers. Butyrate is especially relevant to UC because the colon depends heavily on microbial fermentation products and because butyrate-producing anaerobes are often reduced in active disease [6,12]. Its classical role as an epithelial fuel represents only one facet of its importance. SCFAs also regulate epithelial barrier integrity, macrophage and dendritic cell function, regulatory T-cell differentiation, enteroendocrine signaling, and possibly vagal afferent activity [6,13,14,23,59]. Butyrate and other commensal-derived metabolites promote colonic and peripheral regulatory T-cell (Treg) generation [13,14]. This is of particular relevance to UC because mucosal tolerance must restrain immune responses to a dense microbial antigenic load. Butyrate inhibits histone deacetylases, promotes Foxp3 expression, reduces nuclear factor kappa B (NF-κB)-driven inflammatory programs, and supports tight-junction integrity. Through these effects, SCFAs limit microbial ligand translocation and indirectly restrain immune-cell activation and nociceptive signaling.

In UC, SCFA deficiency may have consequences that extend well beyond impaired epithelial energy supply. The best-supported chain links SCFA depletion to impaired epithelial metabolism and regulatory immune tone, increased microbial ligand exposure, and cytokine-driven neural sensitization. Direct proof of this entire sequence in human UC remains limited, and the observed human associations do not establish temporal direction, with receptor- and Treg-level causality remaining mainly experimental. Nonetheless, its individual components are supported by IBD metabolite literature, epithelial and Treg biology, and experimental neuroimmune studies [6,12,13,14,42,43]. SCFA restoration is therefore most likely to be useful when tied to epithelial metabolic recovery and immune tolerance, rather than treated as a nonspecific supplement.

### 4.2. Tryptophan–Indole–AhR Signaling and Serotonin

Tryptophan metabolism connects diet, microbes, epithelial defense, enterochromaffin cells, neural signaling, and immunity. Microbial indole derivatives activate aryl hydrocarbon receptor (AhR) signaling in epithelial cells, innate lymphoid cells, T cells, and myeloid cells, supporting interleukin-22 (IL-22) production, epithelial repair, antimicrobial defense, and mucosal tolerance [9,15]. Impaired microbial tryptophan metabolism may therefore weaken protective epithelial–immune circuits in UC.

The serotonin branch provides a more explicit neuroimmune mechanism. Most peripheral serotonin is produced by enterochromaffin cells, and gut bacteria can regulate host serotonin biosynthesis [23,24]. UC-focused studies further link cholinergic and serotonin-related signals to mucosal immune cells and disease biology [25,26,27,28]. Serotonin influences secretion, motility, pain perception, epithelial function, mast cell activity, and immune cell behavior. In UC, dysregulated serotonin signaling may connect microbial perturbation to urgency, diarrhea, visceral sensitivity, and immune activation. Human UC findings do not support a simple serotonin-excess model, and integrated longitudinal evidence remains incomplete.

### 4.3. Bile Acids

Bile acids are microbial–host signaling molecules. Gut microbes convert primary bile acids into secondary bile acids, which signal through receptors such as farnesoid X receptor (FXR) and G protein-coupled bile acid receptor 1 (TGR5). These pathways regulate epithelial barrier function, antimicrobial peptide expression, macrophage cytokine production, dendritic cell maturation, inflammasome activity, enteroendocrine signaling, and intestinal motility [7,8,16,17,18]. In UC, altered microbial bile acid transformation may reduce anti-inflammatory receptor signaling and disturb epithelial–neural–immune communication. The principal human study of secondary bile acid deficiency was conducted in patients with UC pouches rather than intact-colon UC, and direct neural effects remain untested in intact-colon UC.

Sinha et al. showed that UC pouches had lower secondary bile acids and biosynthetic gene expression, whereas secondary bile acid supplementation reduced inflammation in three mouse models partly via TGR5 [7]. Thus, the human findings are specific to the pouch environment. Evidence at the receptor level is experimental: FXR activation reduced macrophage inflammatory programs and murine colitis [16], and TGR5/GPBAR1 studies linked receptor signaling to epithelial integrity, IL-10-associated macrophage states, and reduced experimental colitis [17,18].

### 4.4. MAMP Sensing and Barrier-Dependent Amplification

MAMPs, including lipopolysaccharide, flagellin, peptidoglycan, and bacterial DNA, are sensed by Toll-like receptors (TLRs), nucleotide-binding oligomerization domain (NOD)-like receptors, and inflammasome-related pathways. Under homeostatic conditions, controlled microbial sensing supports barrier maintenance. In active UC, mucus and epithelial disruption increase the access of microbial products to lamina propria immune cells and potentially to neural and glial elements [10,19,20]. Cytokines produced by macrophages, dendritic cells, epithelial cells, and neutrophils can sensitize enteric and sensory neurons, while neuropeptides and secretory changes further amplify inflammation.

Microbial ligands differ from metabolites because their signaling depends strongly on compartmentalization. In the lumen, these ligands are expected and often tolerated. After mucus depletion, epithelial injury, or crypt barrier disruption, the same ligands can become inflammatory danger signals. This spatial shift is central to UC. Ligands that cross the epithelial barrier can activate macrophages, dendritic cells, and epithelial inflammasome pathways, and can promote neutrophil recruitment. The resulting cytokines, proteases, reactive oxygen species, and lipid mediators can alter neuronal excitability and glial activation. Defined ligands have not yet been spatially linked to neural activation in human UC.

### 4.5. Integrated Assessment of Microbial Signals

Across these signal classes, human UC evidence is strongest for altered metabolite pools, mucus penetrability, biofilms, and selected mucosal mediators, whereas receptor-level mechanisms are defined mainly in animal and cellular systems. The next step is to link microbial function, individual metabolites or ligands, epithelial integrity, immune-cell state, enteroendocrine activity, neural physiology, and symptoms through longitudinal, anatomically matched studies. Intervention studies can then test whether target engagement in tissue precedes changes in clinical outcomes. Together, these microbial inputs acquire clinical relevance when enteroendocrine, neural, and glial circuits translate them into changes in secretion, motility, sensation, and immune regulation.

Figure 2 presents five parallel microbial signaling pathways and their convergence on the UC barrier–immune–neural disease loop.

## 5. Neural and Glial Nodes in UC

Neural and glial nodes provide a mechanistic explanation for the symptom–inflammation mismatch in UC. Vagal and autonomic pathways modulate inflammatory tone; enteric neurons regulate secretion and motility; sensory neurons and mast cells link inflammation to urgency and pain; and enteric glia coordinate barrier repair and local immune–neural communication.

### 5.1. Vagal and Autonomic Regulation

The vagus nerve is a major conduit between visceral sensing and immune regulation. The cholinergic anti-inflammatory pathway suppresses cytokine production in part through α7 nicotinic acetylcholine receptor (α7 nAChR) signaling in macrophages and related immune cells [44,45,46,47,62,63]. Microbial metabolites, enteroendocrine hormones, inflammatory mediators, and stress signals may modulate vagal afferent and efferent activity. In UC, reduced microbial metabolite production and persistent cytokine exposure may attenuate vagal anti-inflammatory tone.

This pathway is clinically relevant because autonomic imbalance may contribute to relapse vulnerability, stress sensitivity, and persistent symptoms [46,62]. Low vagal tone may reduce the capacity to terminate mucosal cytokine responses after microbial or epithelial injury. Conversely, active inflammation can itself disturb autonomic regulation, thereby making causal inference from cross-sectional studies difficult. Longitudinal UC studies should therefore measure heart rate variability, inflammatory biomarkers, mucosal macrophage activation, microbial metabolites, symptom domains, and endoscopic activity before and after intervention. The direction of the autonomic–inflammatory relationship remains uncertain.

Clinical evidence for neuromodulation remains preliminary. The principal adult VNS pilot study enrolled patients with Crohn’s disease rather than UC, and a mixed pediatric proof-of-concept study was small [47,63]. These studies establish feasibility but not efficacy in UC; future trials require sham control, target-engagement measures, and objective mucosal outcomes.

### 5.2. Enteric Nervous System

The enteric nervous system (ENS) is not merely a motility network. It integrates microbial, epithelial, immune, and stress-related signals. Mechanistic evidence indicates that ENS pathways can relay psychological stress to intestinal inflammation [29,55,56]. These findings support clinical observations that stress can aggravate IBD symptoms and may influence disease activity. For UC, the key point is local mucosal signaling: enteric neurons regulate secretion, blood flow, immune recruitment, barrier function, and nociceptive thresholds.

Enteric neurons are anatomically positioned to integrate mucosal inflammation with functional symptoms. Submucosal neurons regulate epithelial secretion, local blood flow, and immune-cell communication, whereas myenteric circuits shape motility and reflex behavior. In UC, rectal and distal colonic inflammation can therefore produce urgency, tenesmus, and altered stool frequency through effects on local reflex circuits. Cytokines, prostaglandins, microbial metabolites, bile acids, and serotonin can all alter neuronal excitability. Once established, altered excitability may persist after partial mucosal healing, contributing to symptom–inflammation mismatch. The directional molecular sequence remains experimental rather than established in human UC.

### 5.3. Sensory Neurons and Neuropeptides

Gut-innervating sensory neurons detect mechanical, chemical, microbial, and inflammatory cues. They release neuropeptides, including calcitonin gene-related peptide (CGRP), substance P, vasoactive intestinal peptide (VIP), and neuropeptide Y-related signals, that regulate vascular permeability, secretion, immune-cell recruitment, mast-cell activation, and pain transmission [30,32,33,34,35,36]. A key mechanistic study showed that gut-innervating nociceptors can regulate intestinal microbiota to promote tissue protection, demonstrating bidirectional neural–microbial communication [30]. UC-related data linking the microbiome to colonic neuropeptide Y expression further support this axis [32].

Sensory neurons are especially relevant to UC because they bridge mucosal inflammation and patient-centered symptoms. transient receptor potential (TRP) channels, purinergic receptors, cytokine receptors, prostaglandin pathways, and neurotrophic signals can lower activation thresholds during inflammation [30,31,32,33,34,35,36,48]. Once sensitized, sensory neurons can generate pain, urgency, and reflex secretion in response to stimuli that would otherwise be tolerated. Human UC evidence linking residual symptoms to visceral hypersensitivity and mast cell activity supports this clinical relevance, although the microbial upstream drivers remain incompletely defined [31]. The human sensory niche has not yet been linked to a specific microbial trigger.

### 5.4. Enteric Glial Cells

Enteric glial cells regulate epithelial barrier integrity, neuronal survival, immune-cell recruitment, and tissue repair. They respond to microbial products and inflammatory cytokines and release mediators that shape epithelial and immune responses. Experimental UC-related work suggests that therapeutic modulation of enteric glial cell–intestinal epithelial cell–immune cell interactions can ameliorate inflammation [37,38,55]. Broader colitis and enteric glia studies support both protective and reactive glial roles [37,38,39,40,55]. Human findings differ between the mucosal and myenteric compartments.

Enteric glia are increasingly recognized as active mucosal regulators rather than passive support cells. They sense inflammatory mediators, communicate with epithelial cells, modulate neuronal survival, and influence immune-cell positioning [37,38,39,40,55]. Glial mediators may support tight-junction integrity, epithelial restitution, antimicrobial defense, and resolution programs. Persistent glial activation, however, may also produce inflammatory mediators, amplify neuronal excitability, or signal ongoing barrier stress. This dual role is important in UC, where repeated cycles of injury and repair may lead to repeated activation of glial programs. Selective glial causality and therapeutic targeting remain unproven in human UC.

### 5.5. Integrated Evidence Assessment and Priorities

Human UC studies have documented autonomic associations, enteric neuronal remodeling, neuropeptide changes, mast cell–nerve proximity, rectal hypersensitivity, and compartment-dependent glial responses. Evidence for causal circuits remains largely experimental, and routine biopsies do not adequately sample deeper enteric networks. Priority studies should integrate disease activity, symptoms, autonomic and sensory measures, and spatially matched tissue analyses. Thus, neural and glial pathways operate within the local immune and stromal context of mucosal niches.

## 6. Immune-Cell Integration

Immune cells are the central decision points through which microbial and neural inputs are translated into either persistent inflammation or mucosal repair. In UC, these cells cannot be reduced to isolated cytokine producers. Rather, they are embedded in spatial niches containing epithelial cells, microbial products, metabolites, nerves, glia, blood vessels, and stromal cells. A given immune cell may therefore behave differently depending on its exposure to butyrate, indole derivatives, bile acids, acetylcholine, serotonin, neuropeptides, barrier-derived alarmins, or microbial ligands.

### 6.1. Macrophages and Dendritic Cells

Macrophages and dendritic cells integrate microbial ligands, metabolites, epithelial injury signals, and neural mediators. In a tolerant mucosa, resident macrophages clear microbial products while limiting excessive cytokine release. In active UC, barrier disruption increases exposure to bacterial ligands and damage-associated signals, promoting tumor necrosis factor (TNF), IL-1β, IL-6, IL-23, chemokines, and other inflammatory mediators [21,57,64,65]. These mediators recruit additional immune cells and sensitize neurons, creating a feed-forward loop between microbial exposure, myeloid activation, and neural excitability. Human studies establish myeloid remodeling but not the complete microbial–neural–myeloid sequence. A small UC tissue study detected Langerin-positive dendritic cells expressing serotonin and the vesicular acetylcholine transporter [25], identifying a candidate neurochemical interface whose effects on antigen presentation and T-cell priming remain to be defined.

Microbial metabolites and neural signals can modulate this myeloid response. SCFAs restrain inflammatory macrophage programs and support regulatory dendritic cell functions. AhR ligands influence myeloid–epithelial repair pathways. Bile acid receptor signaling shapes macrophage cytokine output and inflammasome activity. Acetylcholine suppresses macrophage cytokine release through cholinergic anti-inflammatory mechanisms, whereas substance P and other neuropeptides enhance vascular permeability and leukocyte recruitment in selected contexts [8,9,13,14,15,16,17,18,19,20,25,27,29,32,33,34,40,44,45,46,47,62,63]. Directional regulation by neural and glial signals is supported chiefly by experimental evidence.

### 6.2. T Cells and Innate Lymphoid Cells

T cells and innate lymphoid cells (ILCs) translate microbial and epithelial signals into tolerance, repair, or chronic effector inflammation. SCFAs support Treg differentiation and function, thereby restraining responses to commensal antigens [13,14,57,58]. Tryptophan-derived AhR ligands support IL-22-associated epithelial repair and influence T-cell and ILC programs [9,15]. When these microbial metabolite pathways are impaired, the mucosa may shift toward excessive effector responses, impaired epithelial repair, and persistent antigen-driven activation. Matched measurements of microbial, neural, and lymphoid parameters remain needed. However, many studies have assessed lymphoid changes without parallel measurements of microbial metabolites or neuronal and glial activity [57,58], leaving their upstream neuroimmune regulation as a testable component of the network.

### 6.3. Mast Cells and Neutrophils

Mast cells connect mucosal immune activation with pain, urgency, and barrier dysfunction. They respond to microbial products, epithelial alarmins, stress-related mediators, neuropeptides, and serotonin. Once activated, they release histamine, proteases, cytokines, and lipid mediators that increase epithelial permeability and sensitize nearby sensory neurons. In patients with UC in remission, persistent symptoms have been associated with visceral hypersensitivity and mast cell activity, supporting the clinical plausibility of a mast cell–nerve axis [31]. These observations support a sensory association without establishing a microbial cause.

Neutrophils are central to active UC pathology and crypt abscess formation. Their recruitment reflects barrier breakdown and strong chemokine signaling, but their products can also influence neural and epithelial function. Proteases, reactive oxygen species, antimicrobial peptides, and extracellular traps may contribute to tissue injury and expose additional microbial ligands. In a microbiota–neuroimmune framework, neutrophils are not merely terminal inflammatory effectors; they amplify barrier damage that sustains microbial access to immune and neural structures. Human UC and experimental evidence for neutrophil extracellular trap (NET) amplification are summarized in reference [22].

### 6.4. Spatial Immune Niches

A biopsy showing increased inflammatory transcripts does not reveal whether macrophages are located near breached crypts, mast cells are adjacent to sensory fibers, glial activation surrounds damaged epithelium, or Treg/ILC repair programs are present at ulcer margins. These relationships are essential for understanding microbiota-driven neuroimmune crosstalk. Single-cell and spatially resolved IBD platforms now make this type of mapping feasible, but direct UC datasets integrating microbiota, metabolites, nerves, glia, and immune-cell niches remain rare [57,64,65]. Current spatial studies resolve immune and stromal organization more clearly than they resolve microbiota–neural–immune integration [57,64,65].

**Figure 3:**
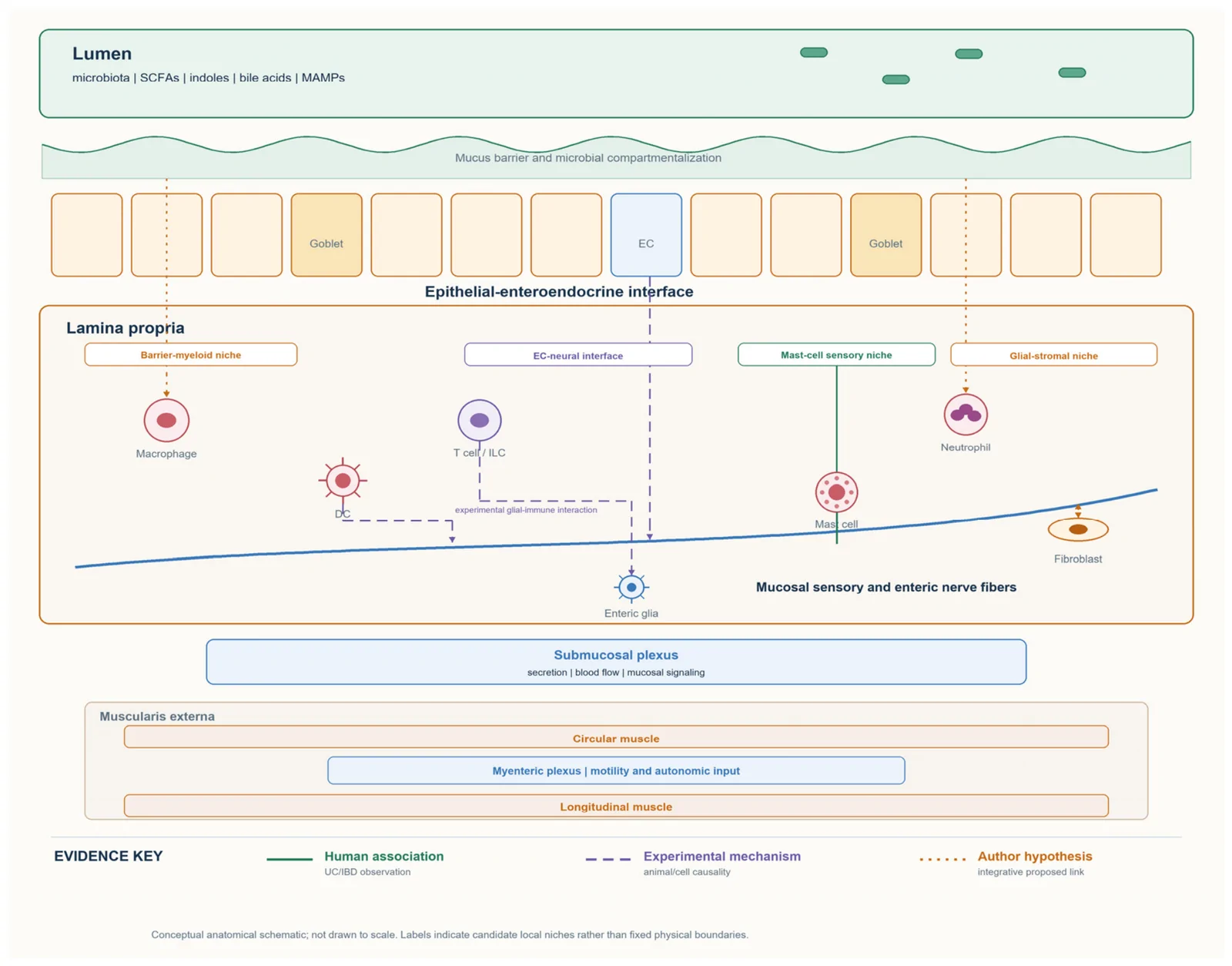
Spatial organization of candidate neuroimmune mechanisms across colonic wall layers in UC. Luminal metabolites and MAMPs first encounter the mucus and epithelial–enteroendocrine interface. Barrier failure increases access to macrophages and dendritic cells; enterochromaffin mediators connect luminal sensing to mucosal nerve fibers; mast cell proximity can amplify sensory signaling; and enteric glia link neural, epithelial, immune, and stromal responses. The submucosal and myenteric plexuses provide routes through which inflammation can alter secretion, blood flow, motility, and autonomic input. Green solid lines indicate human associations, purple dashed arrows indicate experimental mechanisms, and orange dotted arrows indicate proposed relationships. The T cell/ILC-glial connector denotes an experimental glial–immune interaction [29,38]. The drawing is conceptual and not to scale. Abbreviations: DC, dendritic cell; EC, enterochromaffin; IBD, inflammatory bowel disease; ILC, innate lymphoid cell; MAMP, microbe-associated molecular pattern; SCFA, short-chain fatty acid; UC, ulcerative colitis.

The most useful endpoints will combine cell states, spatial proximity, metabolite exposure, neural and glial markers, and clinical domains such as urgency, pain, endoscopic activity, and relapse. Spatially matched microbial exposure, epithelial integrity, immune state, and neural or glial markers are needed to define the candidate niches shown in Figure 3, which provides the anatomical counterpart to the system-level network by locating candidate neuroimmune niches across the colonic wall.

## 7. UC-Specific Human Evidence and Integrated Disease Model

A UC-focused review must distinguish disease-specific human evidence from broader IBD or experimental colitis data. Direct human UC data are strongest for clinical phenotype, mucosal inflammation, epithelial injury, microbiota shifts, fecal metabolites, and responses to selected microbiota-directed interventions—establishing the clinical relevance of the microbial and immune components of the proposed axis [1,4,5,6,7,10,11,12,19,21,22,23,24,25,27,31,32,37,46,51,52,53,55,56,57,58,60,61,64,65,66,67,68,69,70]. Evidence is also accumulating for neuroactive changes, including microbiome-associated colonic neuropeptide expression and symptom-related neural pathways [31,32,35,36,48]. Direct human UC evidence that simultaneously links microbiota, neurons, glia, and immune-cell states within the same tissue region remains limited.

Building on this evidence base, we propose an author-derived, hypothesis-generating four-phase temporal model rather than an established natural history. The first phase is variable mucosal resilience during apparent clinical quiescence. A 13-patient recovery-phase cohort sampled previously inflamed sites after recent treatment for relapse identified increased epithelial permeability and mucosal immune abnormalities, while separate fecal microbial and multi-omics studies associated baseline features with differential subsequent relapse risk [66,67,68]. These findings reveal residual biological heterogeneity; the associations do not assign a causal role to the measured features, and the permeability result may not generalize to stable long-term remission [66,67,68].

The second phase comprises perturbation and a shift in the neuroimmune threshold. Experimental evidence indicates that chronic stress can alter enteric glial and neuronal states and increase monocyte-dependent inflammation, providing a possible mechanism by which a systemic perturbation could change local inflammatory susceptibility [29]. Such neural changes may interact with pre-existing barrier weakness or microbial–metabolic instability, although their temporal order in human UC is unknown.

The third phase is mucosal amplification. Active UC is characterized by inflammatory monocyte and macrophage programs, IL-17-associated T-cell remodeling, spatial myeloid–stromal interactions, and NET-associated inflammatory activity [21,22,57,58,64]. When these processes coexist, epithelial damage could increase exposure to luminal material, while inflammatory mediators alter stromal, neuronal, and immune behavior and sustain a feedback loop.

The fourth phase is divergent recovery after inflammatory control and reframes residual symptoms. Persistent urgency or pain after mucosal improvement should not be dismissed as purely functional, nor automatically treated as occult inflammation. It may represent a post-inflammatory neuroimmune state involving sensory neuron sensitization, mast cell activation, serotonin signaling, altered motility, and microbial metabolite deficits. Conversely, persistent symptoms may also reflect incomplete mucosal healing. Distinguishing these possibilities requires combined inflammatory, microbial, metabolic, and neuroimmune biomarkers rather than escalation of immunosuppression based on symptoms alone. Persistent symptoms still require objective assessment because inflammation, hypersensitivity, and mast cell–nerve activity can overlap [31,48,69,70].

This model links causality, context, and timing: similar symptoms may arise from different combinations of inflammation, barrier dysfunction, sensory sensitization, and microbial–metabolic instability, while a single measured feature may be a driver, consequence, or compensatory response. Longitudinal flare-to-remission sampling is needed to distinguish these roles.

## 8. Biomarkers and Hypothesis-Generating Endotypes

### 8.1. Multimodal Biomarker Framework

A major challenge is that standard UC endpoints do not directly capture neuroimmune state. Symptoms, C-reactive protein (CRP), fecal calprotectin, endoscopy, and histology remain essential, but they do not measure vagal tone, sensory neuron sensitization, enteric glial activation, serotonin signaling, or microbial metabolite function. Candidate biomarkers include stool metagenomics, strain-level profiling, short-chain fatty acids (SCFAs), indole derivatives, kynurenine metabolites, bile acids, microbial ligands, mucins, tight-junction proteins, epithelial stress markers, macrophage states, Treg/Th17 balance, ILC3–IL-22 pathways, mast cell signatures, neuropeptides, serotonin pathway enzymes, glial fibrillary acidic protein (GFAP), S100B, and autonomic markers. No single component is currently validated as a stand-alone microbiota–neuroimmune biomarker.

A barrier and spatial domain could combine epithelial permeability with the localization of myeloid cells, fibroblasts, mast cells, and nerve-associated structures. Increased colonic permeability and mucosal immune-cell abnormalities were reported in a small recovery-phase UC cohort, whereas spatial studies resolved macrophage–neutrophil heterogeneity and mononuclear phagocyte–fibroblast proximity in inflamed tissue [64,65,66]. Mast cell proximity to nerve endings is a candidate feature of a symptom-associated niche, but current support comes from a small remission cohort [31].

A microbial–metabolic domain can prioritize longitudinal function over a single taxonomic signature. Fecal microbial clusters were associated with different clinical courses in quiescent UC, but some individual taxa showed directions that would not be predicted from conventional labels of beneficial or harmful bacteria [67]. In a mixed quiescent IBD cohort, relapse was associated with proteomic markers including IL-10, glial cell line-derived neurotrophic factor (GDNF), and CD8α, metabolomic markers including propionyl-L-carnitine, carnitine, sarcosine, and sorbitol, and increased abundance of selected microbial groups [68]. These signals require replication because the cohort contained a minority of patients with UC and few relapse events [68].

A neurofunctional and symptom domain could include validated symptom instruments together with rectal sensory testing and psychological measures. Visceral hypersensitivity was associated with symptom severity in quiescent UC, and irritable bowel syndrome (IBS)-like symptoms remained frequent in a population-based cohort even among patients in deep remission [48,69]. Enteric glial inflammatory states and glial–nociceptor signaling are mechanistically informative experimental candidates, but they have not yet yielded validated human UC biomarkers [29,39]. Symptom scores cannot substitute for direct measures of inflammation, sensory physiology, or neural and glial state.

### 8.2. Hypothesis-Generating Endotypes

These proposed endotypes are not yet validated clinical categories. They provide a framework for prospective cohort stratification and biomarker studies, but they should not currently guide diagnosis or treatment selection. The four groupings below are overlapping, potentially time-varying, and hypothesis-generating research constructs.

**Barrier–myeloid amplification.** Epithelial permeability or injury, inflammatory monocyte or macrophage programs, NET activity, and spatial myeloid–fibroblast organization can be evaluated together [21,22,64,66]. Dendritic cell serotonin or cholinergic markers remain exploratory features [25].

**IL-17/ILC remodeling.** Effector and regulatory T-cell states, type 3 innate lymphoid cell (ILC3) abundance, and their spatial relationships with epithelial, myeloid, and stromal populations can be assessed together [57,58]. Cohort studies can test how microbial metabolism, cytokine exposure, and treatment shape this pattern.

**Relapse vulnerability.** Replicated barrier, microbial, proteomic, or metabolomic features may identify residual biological vulnerability during clinical quiescence [66,67,68]. Current studies provide associations and risk discrimination rather than a resolved causal mechanism.

**Post-inflammatory sensory dysfunction.** Persistent symptoms after objective inflammatory control can be studied through rectal hypersensitivity, mast cell activation or nerve proximity, and experimental markers of enteric glial signaling [31,39,48,69]. One remission study found no significant fecal compositional difference between symptomatic and asymptomatic UC groups [70].

These groupings may overlap and change across flare, treatment, and remission. Prospective studies should link longitudinal stool, metabolite, blood, and anatomically matched biopsy measurements with endoscopic, histologic, sensory, and symptom outcomes. Their value lies in generating testable links among biological measurements, symptoms, relapse risk, and treatment response rather than in assigning fixed labels.

## 9. Therapeutic Opportunities

The therapeutic implication of this framework is that microbiota-directed and neural interventions should be judged by target engagement within the UC disease circuit, not by taxonomic change or symptom improvement alone. A credible intervention should specify whether it aims to restore a microbial substrate or metabolite, introduce a defined strain or community, reduce barrier-dependent microbial signaling, or modulate enteroendocrine, sensory, or autonomic pathways. Human evidence is intervention-specific: defined fiber or prebiotic preparations [71,72], probiotic formulations [49,50], one synbiotic combination [73], SCFA or butyrate preparations [42,43,74], and selected FMT protocols [51,52,53,60,61,75] have been studied in UC. VNS evidence remains preliminary and is derived from Crohn’s disease or mixed pediatric IBD studies rather than from an adequately powered UC efficacy trial [47,63,76]. Reference [77] provides the consensus postbiotic definition, but no qualifying randomized UC postbiotic trial was identified among the studies included in this review. Guideline-based UC treatment and trial restrictions remain applicable [3,54,78]. Table 4 summarizes the corresponding clinical boundaries.

Across modalities, evidentiary depth is uneven. Randomized UC evidence exists for selected fiber preparations, strain- or formulation-specific probiotics, SCFA preparations, and protocol-specific FMT, but results cannot be generalized across products, strains, donors, routes, or disease states. Synbiotic evidence remains pilot-level; among the studies included in this narrative review, no qualifying randomized UC postbiotic trial was identified; and VNS, serotonin, and neuropeptide strategies remain unproven as UC therapies. Thus, no class-wide “microbiome therapy” effect is justified, and each intervention should be evaluated at the level of the tested product or protocol, with objective outcomes and mechanism-specific target engagement.

### 9.1. Diet, Fiber, and Prebiotics

Fiber and prebiotics act upstream by changing substrate availability, fermentation, luminal pH, and the ecological fitness of metabolite-producing organisms. The mechanistic aim is to restore outputs such as SCFAs that support colonocyte metabolism, tight-junction integrity, Treg differentiation, and enteroendocrine signaling [6,13,14,23,59]. In an open-label randomized study in remission, 12-month treatment-failure rates were 40% with *Plantago ovata*, 35% with mesalamine, and 30% with combination treatment, without a statistically significant between-group difference; because this was not a definitive equivalence or noninferiority trial, it does not establish Plantago as a replacement for mesalamine [71]. A small fructo-oligosaccharide (FOS)-enriched inulin pilot showed tolerability and an early fecal calprotectin reduction but was not powered for clinical or endoscopic remission [72]. These trials tested defined fiber preparations rather than whole-diet patterns. Future studies should specify substrate, dose, background diet, disease activity, tolerability, microbial function, metabolite target engagement, and objective mucosal outcomes.

### 9.2. Probiotics

Probiotics could act through transient ecological competition, bacteriocin production, reinforcement of epithelial defenses, altered metabolite output, or modulation of innate and adaptive immunity. These mechanisms cannot be inferred from the label “probiotic” alone: strain identity, viability, dose, manufacturing, delivery matrix, and compatibility with the recipient ecosystem determine biological activity. Escherichia coli Nissle 1917 showed maintenance outcomes similar to mesalamine in one double-blind trial, and a defined multistrain preparation improved response and remission in mild-to-moderately active UC [49,50]. However, the efficacy of one strain or mixture cannot be generalized to another, and compositional detection does not establish mucosal target engagement. Current American Gastroenterological Association (AGA) guidance restricts probiotic use in UC to clinical-trial settings [78]. Future studies should verify product identity and stability, quantify strain persistence and functional output, maintain stable background therapy, and pair clinical and endoscopic endpoints with barrier, metabolite, and immune measures.

### 9.3. Synbiotics

Synbiotics combine a live microorganism with a substrate intended to be selectively used by that organism or by a beneficial microbial network. Their mechanistic advantage is potential ecological cooperation: the substrate may improve engraftment or metabolic activity, while the organism may convert it into SCFAs or other products that influence epithelial and immune programs. Their central interpretive problem is attribution. In an 18-patient pilot trial, Bifidobacterium longum with inulin-oligofructose reduced selected mucosal inflammatory markers and histologic inflammation, although the sigmoidoscopy difference was not statistically significant [73]. This small study cannot determine whether the organism, substrate, interaction, or background treatment produced the signal. Adequately powered factorial trials should therefore compare each component and the combination, document substrate utilization and strain persistence, and test whether metabolite and mucosal changes mediate clinical benefit.

### 9.4. Postbiotics

Using the consensus definition, a postbiotic is a preparation of inanimate microorganisms and/or their components that confers a health benefit [77]. Potential advantages include stability and reduced replication risk, whereas inactivation may destroy activity and retained MAMPs may be harmful when the barrier is breached. Among studies included in this narrative review, no randomized UC trial met the consensus definition; purified metabolites such as butyrate are considered separately. Initial UC studies should report source organism, inactivation method, retained components, batch consistency, biological potency, mucosal safety, background therapy, and a pre-specified target-engagement marker.

### 9.5. Butyrate and Other Short-Chain Fatty Acids

SCFAs link microbial fermentation to colonocyte energy metabolism, histone deacetylase inhibition, free fatty acid receptor signaling, Treg differentiation, barrier integrity, and enteroendocrine activity [6,13,14,23,59]. Direct supplementation, however, bypasses only the metabolite deficit and does not necessarily correct the ecological, barrier, or inflammatory conditions that produced it. Delivery is also critical because luminal concentration, disease location, epithelial uptake, retention time, and background inflammation determine mucosal exposure. Nightly butyrate enemas did not improve clinical outcomes relative to placebo in distal UC, and rectal SCFA irrigation did not provide a significant overall benefit in a larger randomized trial, although exploratory subgroup analyses suggested possible effects [42,43]. A small oral sodium-butyrate trial reported reductions in fecal calprotectin and high-sensitivity C-reactive protein and improvements in sleep and quality-of-life measures, but it was not a definitive remission trial [74]. Current evidence therefore does not support routine SCFA products as replacements for standard therapy. Mechanism-based studies should stratify baseline fermentation capacity and epithelial competence and demonstrate exposure, receptor or metabolic engagement, and mucosal benefit.

### 9.6. Fecal Microbiota Transplantation

FMT is the most direct attempt to alter a microbial community and its collective metabolic capacity. Its plausible actions include displacement of inflammation-associated communities, restoration of anaerobic functions, altered bile acid and SCFA metabolism, and reduced pro-inflammatory ligand exposure. Selected intensive, multidonor, and oral protocols have provided randomized proof of concept for short-term remission [51,52,60,75], whereas a trial using two nasoduodenal administrations was negative [53]. An FMT-plus-diet study supported an integrated strategy but could not isolate the contribution of each component [61]. These divergent results show that FMT is not a single intervention: donor selection, processing, oxygen exposure, route, dose, schedule, comparator, antibiotics, diet, recipient disease state, and background therapy can all alter efficacy. Conventional FMT remains restricted to clinical trials in IBD [54]. Trials should combine rigorous donor and pathogen screening with batch-level characterization, functional metabolomics, engraftment analyses, objective mucosal outcomes, and long-term assessment of infection, ecological stability, and durability.

### 9.7. Vagus Nerve Stimulation

Vagus nerve stimulation (VNS) targets a host regulatory circuit rather than the microbiota directly. Cholinergic signaling can suppress macrophage cytokine production, while vagal activity may integrate microbial, enteroendocrine, stress, and inflammatory signals [44,45,46,62]. Clinical evidence remains preliminary. The principal adult implant study enrolled patients with Crohn’s disease [47]. A 22-participant pediatric proof-of-concept study included 12 patients with UC and used a 2-week randomized sham-controlled crossover followed by open-label stimulation, but it was not designed to establish UC-specific efficacy or mucosal healing [63]. No adequately powered parallel-group sham-controlled trial has established VNS efficacy in UC. Future trials require standardized stimulation, autonomic target engagement, objective mucosal outcomes, symptom measures, and device-specific safety monitoring [76].

### 9.8. Serotonin and Neuropeptide Pathways

Serotonin and neuropeptides are receptor-, cell-, compartment-, and disease-stage-dependent therapeutic candidates. Microbial and SCFA signals can regulate enterochromaffin cell serotonin biosynthesis, while human UC tissue shows altered serotonin pathway markers and neurochemical features in dendritic cells [23,24,25,26,27,28]. Sensory mediators such as substance P, CGRP, VIP, and NPY can influence vascular permeability, secretion, immune recruitment, nociception, and tissue protection, and UC studies support altered neuropeptide expression and mast cell–nerve associations [30,31,32,33,34,35,36]. These findings do not justify broad suppression or enhancement of an entire pathway because the same mediator may exert protective and inflammatory effects through different receptors or cells. Early clinical studies should select a defined receptor and phenotype, distinguish symptom control from disease modification, and measure urgency, pain, secretion, motility, inflammatory activity, and safety separately.

### 9.9. Combination Strategies

**Figure 4:**
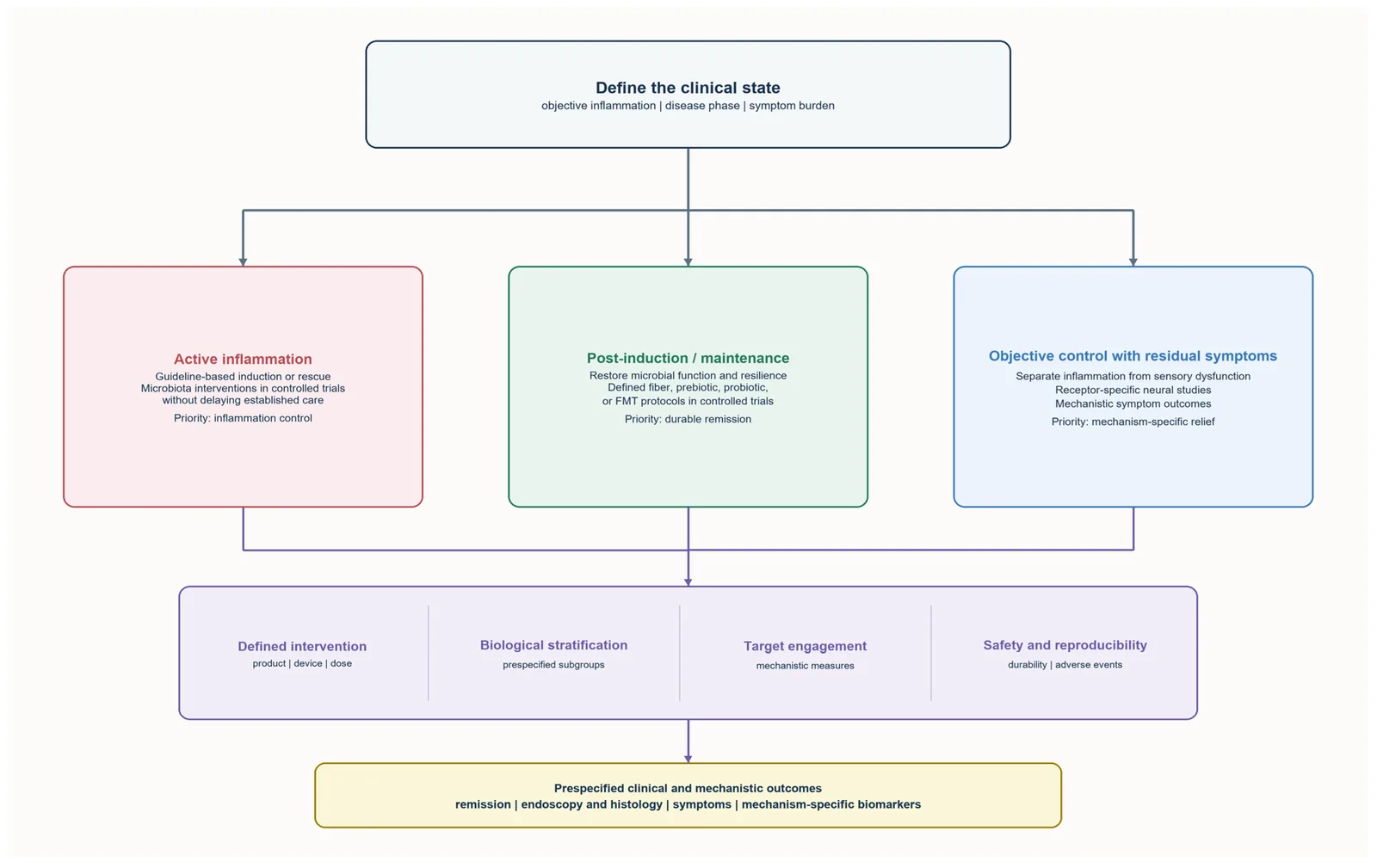
Clinical-state roadmap for mechanism-linked trials. Objective assessment distinguishes active inflammation, post-induction or maintenance, and residual symptoms after inflammatory control. The intervention should then be matched to a specified mechanism: immune control during active disease; restoration of microbial function and barrier resilience after induction; or receptor- and phenotype-specific neural evaluation after inflammation is controlled. Across these settings, trials should define the product or device, population, biological strata, target-engagement measures, clinical and mechanistic outcomes, and duration of safety follow-up. Abbreviation: FMT, fecal microbiota transplantation.

Combination strategies should be staged according to the dominant, objectively measured disease process rather than applied as an undifferentiated microbiome package. During severe active inflammation, rapid guideline-based immune control and rescue planning take priority; microbiota-directed approaches, if studied, should be adjunctive and should not delay effective treatment. After induction, defined dietary, prebiotic, probiotic, synbiotic, metabolite, or FMT protocols could be tested for restoration of microbial function and barrier resilience. After objective inflammatory control, persistent urgency or pain should prompt assessment of residual inflammation, sensory hypersensitivity, mast cell activity, motility, and psychological factors before neural or symptom-directed interventions are selected. Autonomic dysfunction may identify a research subgroup for neuromodulation, but not a validated treatment endotype. Factorial or multi-arm designs with stable background therapy are needed to determine whether combinations are additive, synergistic, or redundant and whether target engagement mediates clinical benefit. Figure 4 translates these principles into a clinical-state trial roadmap.

Figure 4 previews the clinical-state and trial-design principles developed in Section 10.

## 10. Research and Trial Design Roadmap

### 10.1. Biological and Clinical Stratification

For research design, microbiota–neuroimmune mechanisms should not be inferred from a single data layer. Microbial profiling is more informative when paired with metabolomics, and metabolite data require mucosal immune and epithelial context. Studies of urgency, pain, or autonomic function should include microbial and metabolic measures to distinguish upstream drivers from symptom correlates. In trials, mechanistic biomarkers are needed to show whether response reflects restored microbial metabolism, reduced neural sensitization, barrier repair, immune suppression, or combined effects. These variables should be pre-specified rather than selected retrospectively from responders.

Microbiota-directed interventions require enrichment variables aligned with their mechanisms. Low fermentation capacity or low fecal short-chain fatty acid (SCFA) concentrations may inform fiber and butyrate studies, whereas baseline strain compatibility is more relevant to probiotic and synbiotic studies. Fecal microbiota transplantation (FMT) studies can assess donor–recipient ecological features without assuming that any single taxonomic profile predicts response.

Neural interventions call for a distinct set of markers. Autonomic dysfunction may help characterize vagus nerve stimulation (VNS) populations, while enterochromaffin cell, 5-hydroxytryptamine (5-HT) receptor, neuropeptide, mast cell, or sensory signatures may inform studies of specific neural pathways. Across intervention classes, stratification variables are most informative when incorporated prospectively into randomization and analysis rather than selected retrospectively from responders.

### 10.2. Therapeutic Timing

Trial timing is also important. Severe active UC may be dominated by acute inflammatory injury, bleeding, epithelial ulceration, and the urgent need for immune suppression. In that context, subtle microbiota–neuroimmune interventions may have limited stand-alone efficacy. Remission maintenance, post-induction consolidation, residual symptom management, and relapse prevention may be more suitable contexts for restoring microbial and neural homeostasis. This distinction may explain why microbiota interventions show variable effects across studies: the same intervention may be poorly suited for severe active inflammation but useful for rebuilding resilience after inflammatory control. Active-disease studies must not delay guideline-based induction or established rescue pathways [3].

### 10.3. Clinical and Mechanistic Endpoints

Conventional UC trials usually prioritize clinical remission, endoscopic improvement, histologic remission, and inflammatory biomarkers. These endpoints are essential but insufficient for microbiota–neuroimmune interventions. If a therapy is proposed to act through microbial metabolites, neural circuits, or glial–immune communication, trials should include endpoints that reflect those pathways. For microbiota-directed therapy, this means measuring not only taxonomic change but also functional gene pathways, metabolite output, bile acid transformation, tryptophan metabolism, microbial ligand burden, and mucosal immune responses. For neuromodulation, this means measuring autonomic tone, vagal markers, cytokines, macrophage states, symptom domains, and microbial metabolite changes. Mechanistic endpoints should be pre-specified and linked to the intervention’s stated mode of action; changes in composition, autonomic measures, or symptoms alone do not demonstrate disease modification.

### 10.4. Longitudinal and Compartment-Matched Sampling

The first conceptual challenge is causality. Dysbiosis may cause inflammation, result from inflammation, or both. Neural sensitization may amplify inflammation, arise from inflammation, or persist after inflammation improves. Microbial metabolites may protect the mucosa under homeostatic conditions but have different effects during epithelial ulceration. These bidirectional relationships make cross-sectional studies difficult to interpret. Longitudinal designs before flare, during active disease, after induction therapy, and during remission are needed to distinguish cause, consequence, and compensation.

### 10.5. Causal Testing

Associations among taxa, metabolites, neural markers, and clinical response require temporal and functional testing. Pre-specified mediation analyses can contribute to inference, while factorial designs can separate diet from FMT and probiotic from prebiotic effects. Dose–response or withdrawal–rechallenge designs may add support when ethically acceptable, and patient-derived organoid, immune-cell, neuronal, or glial systems can test whether candidate organisms or metabolites reproduce human signals. Mechanistic inference will be strongest when intervention effects, temporal ordering, target engagement, and functional validation converge.

### 10.6. Safety and Reproducibility

The fourth challenge is translation. Microbiota and neural interventions require safety assessment specific to their biological and technical risks. Physiological pathways, however, can be harmful when activated in the wrong context. FMT carries donor, infection, and durability concerns. Probiotic effects are strain-specific and cannot be generalized. VNS requires optimization of dose, site, duration, and patient selection. Serotonin or neuropeptide modulation may improve symptoms but could also affect motility, repair, vascular tone, or immune defense. Rigorous trials are therefore essential before these approaches can be positioned as UC therapies. Publicly available protocols, negative findings, and batch- or device-level intervention characteristics will be essential for reproducibility [54,76,77].

## 11. Conclusions

This perspective bridges basic mucosal neurobiology and clinical UC management, emphasizes measurable intermediates rather than speculative pathways alone, and clarifies where evidence is strong enough for translational testing versus where it remains hypothesis-generating. By focusing on signal transduction across compartments, the microbiota–neuroimmune model can help organize a field that otherwise risks fragmentation into separate studies on the microbiome, immunology, neurology, and symptoms.

UC pathogenesis cannot be fully explained by dysbiosis or immune activation considered in isolation. The gut microbiota may shape neuroimmune crosstalk through metabolites, microbial ligands, epithelial barrier signals, enterochromaffin cells, enteric neurons, vagal pathways, sensory neurons, enteric glia, and immune-cell networks. This axis provides an organizing framework for the coexistence of mucosal inflammation, pain, urgency, relapse, stress sensitivity, and variable treatment responses. The next stage of research should prioritize causal human evidence, UC-specific neuroimmune atlases, functional microbiome–metabolome integration, and mechanism-linked trials that evaluate microbiota-directed, neural, and immune-targeted strategies. Until such validation is available, microbiota–neuroimmune interventions should be presented as rational and testable strategies rather than substitutes for guideline-based UC therapy. The proposed endotypes are author-derived, overlapping, potentially time-varying, and hypothesis-generating; they are not validated clinical classifications and should not guide current diagnosis or treatment selection.

None of the priority questions in Table 1 is resolved by current evidence. Priorities are to test whether restoring fermentation improves barrier, immune, and neural outcomes in the same patients; reproduce metabolite and receptor states across UC cohorts; link intact-colon bile acid profiles to macrophage, enteroendocrine, and motility phenotypes; establish whether barrier failure precedes neural or glial activation during flare; conduct adequately powered sham-controlled UC neuromodulation trials; distinguish sensory-mast cell and glial states associated with symptoms, active inflammation, repair, or persistence; validate functional biomarkers of response to defined microbiota-directed interventions; and determine whether the proposed endotypes reproduce longitudinally and relate to relapse, symptoms, or treatment response. These are research priorities, not current clinical recommendations.

Ultimately, the next step is to test these relationships longitudinally in biologically stratified patients and to determine whether restoring microbial function, barrier integrity, immune regulation, or neural stability improves both objective inflammation and patient-reported symptoms alongside established UC therapy.

## Figures and Tables

**Table 1:** Evidence context and unresolved questions by mechanism module.

Module	Evidence Context	Current Limitation	Priority Question
SCFAs	Level B human UC association plus Levels D/E causal mechanisms [6,13,14]	Direct supplementation trials are inconsistent and rarely include neural endpoints [42,43]	Does restoring fermentation improve barrier, immune, and neural outcomes in the same patients?
Tryptophan, indoles, AhR, and 5-HT	Levels C–E support microbial regulation; human UC evidence is mainly cross-sectional [9,15,27]	Human UC evidence is cross-sectional; the integrated metabolite–neural sequence awaits longitudinal testing	Which measured metabolite and receptor states identify reproducible UC phenotypes?
Bile acids	Level C intact-colon IBD evidence and Level B UC-pouch evidence, with Levels D/E receptor mechanisms [7,8]	Pouch anatomy and mixed-IBD cohorts limit extrapolation to intact-colon UC	Can intact-colon UC bile-acid profiles be linked prospectively to macrophage, enteroendocrine, and motility states?
MAMPs and barrier access	Level B evidence supports abnormal mucus and biofilm organization; neural/glial causality is experimental [10,19,20]	Spatial links between defined microbial ligands and neural activation remain to be tested in human UC	Does barrier failure precede neural/glial activation during a flare?
Vagal and autonomic pathways	Strong Level E/D anti-inflammatory biology and Level B autonomic associations [44,45,46]	The principal adult VNS pilot involved Crohn’s disease; randomized UC evidence is still needed [47]	Can sham-controlled UC trials show autonomic target engagement and objective mucosal benefit?
Sensory neurons and mast-cell niches	Level D circuit evidence plus Level B UC remission studies [30,31,48]	Human studies have not yet defined the microbial drivers or receptor-specific sensory effects	Which spatial sensory-mast-cell features distinguish residual symptoms from inflammation?
Enteric glia	Levels B/D/E show reactive human glial changes and experimental epithelial–immune interactions [37,38,39]	Mucosal and myenteric glia are sampled differently, and longitudinal human biomarkers are needed	Are glial states causes, repair responses, persistent sequelae, or combinations of these?
Microbiota-directed therapy	Level A evidence is strongest for formulation-specific probiotics and selected FMT protocols [49,50,51,52]	Formulation and protocol heterogeneity, null trials, durability, and safety limit broad conclusions [53,54]	Can functional biomarkers prospectively identify responders and mechanism?
Integrated model and endotypes	Level H synthesis of component evidence presented in Section 7 and Section 8 [25,29,31,39,48]	Author-derived, overlapping, potentially time-varying, and not validated for clinical classification	Can prospective multimodal cohorts reproduce these groupings and link them to relapse, symptoms, or treatment response?

Table note: A, direct human UC intervention; B, human UC observational or tissue evidence; C, human IBD evidence not specific to UC; D, in vivo experimental evidence; E, ex vivo, in vitro, or foundational non-UC mechanism; H, author-derived hypothesis. The labels describe study context and inferential distance rather than formal risk of bias. Abbreviations: 5-HT, 5-hydroxytryptamine; AhR, aryl hydrocarbon receptor; FMT, fecal microbiota transplantation; IBD, inflammatory bowel disease; MAMP, microbe-associated molecular pattern; SCFA, short-chain fatty acid; UC, ulcerative colitis; VNS, vagus nerve stimulation.

**Table 2:** Cellular components of microbiota–neuroimmune crosstalk in UC.

Component	Principal Signals/Functions	Supported Interpretation	Evidence Boundary
Enterochromaffin cells	5-HT release; nutrient, SCFA, purinergic, and mechanical sensing	Potential epithelial–neural transducer [23,27,28]	Microbial regulation is strongest in experimental systems; human UC data are cross-sectional
Vagal/autonomic pathways	Cholinergic anti-inflammatory signaling; autonomic regulation	Plausible systemic regulator of mucosal inflammation [44,45,46]	Therapeutic efficacy in UC awaits controlled trials
Enteric neurons	Secretion, motility, vascular tone, local reflexes	Altered architecture and coding occur in severe human UC [55,56]	Resection specimens represent advanced disease and provide limited temporal information
Sensory neurons	TRPV1 pathways; substance P; NPY; mast-cell proximity	Experimental circuit effects and human remission studies support sensory and symptom relevance [30,31,32,48]	Microbial drivers and receptor-specific effects in human UC remain uncertain
Enteric glia	Barrier support, trophic signals, inflammatory mediators	Reactive changes and experimental epithelial–immune interactions [37,38]	Human compartment-resolved longitudinal data are sparse
Macrophages/DCs	Microbial sensing, cytokines, antigen presentation, neurochemical interfaces	UC myeloid reprogramming is well supported [21,25]	The complete microbial–neural–myeloid sequence has not yet been tested in human UC
T cells/ILCs	IL-17-associated states, Treg programs, IL-22-associated repair	Disease-associated lymphoid states occur in UC [57,58]	Matched metabolite, neural, and lymphoid measurements are needed
Mast cells/neutrophils	Histamine, proteases, lipid mediators, NETs	Mast-cell–nerve niches relate to symptoms; NETs amplify active inflammation [22,31]	Mast-cell sensory niches and neutrophil tissue-damage loops represent distinct routes

Table note: Rows distinguish directly observed human UC findings from mechanistic inference. Abbreviations: 5-HT, 5-hydroxytryptamine; DC, dendritic cell; IL, interleukin; ILC, innate lymphoid cell; NET, neutrophil extracellular trap; NPY, neuropeptide Y; SCFA, short-chain fatty acid; TRP, transient receptor potential; Treg, regulatory T cell; UC, ulcerative colitis.

**Table 3:** Evidence provenance and uncertainty for microbial signal classes relevant to UC.

Signal Class	Human Evidence	Experimental Evidence	Key Limitation
SCFAs	Lower fecal SCFAs and selected butyrate-producing taxa in one UC cohort [6]	Treg differentiation and EC-cell serotonin regulation [13,14,23]	The effect of SCFA restoration on neural outcomes has not yet been tested in human UC
Indoles/AhR	Reduced AhR-ligand production reported in mixed IBD evidence [9]	AhR-dependent barrier protection and repair [9,15]	UC-specific longitudinal evidence is currently unavailable
Serotonin-related signals	Reduced mucosal serotonin-pathway markers and dendritic-cell 5-HT/VAChT co-expression have been reported in UC tissue [25,27]	Microbiota and SCFAs regulate host serotonin biosynthesis [23,24]	Direction and receptor effects vary; a simple excess model is inadequate
Bile acids	Altered pools in mixed IBD and the UC-pouch environment [7,8]	FXR/TGR5-linked epithelial and myeloid regulation [16,17,18]	Generalizability to intact-colon UC remains uncertain
MAMPs	Abnormal mucus penetrability and mucosal biofilms in subsets of UC [10,19]	TLR-linked ENS and glial responses [20,40]	Defined ligands have not yet been linked spatially to neural activation in human UC

Table note: Abbreviations: 5-HT, 5-hydroxytryptamine; AhR, aryl hydrocarbon receptor; EC, enterochromaffin; ENS, enteric nervous system; FXR, farnesoid X receptor; IBD, inflammatory bowel disease; MAMP, microbe-associated molecular pattern; SCFA, short-chain fatty acid; TGR5, G protein-coupled bile acid receptor 1; TLR, Toll-like receptor; Treg, regulatory T cell; UC, ulcerative colitis.

**Table 4:** Therapeutic strategies and current evidence boundaries.

Strategy	Human UC Evidence	Current Interpretation	Key Design Requirement
Fiber/prebiotics	Small trials of Plantago and inulin/FOS [71,72]	Adjunctive signals; efficacy depends on substrate, setting, and patient tolerance	Specify substrate, dose, diet, activity, tolerance, and objective endpoints
Probiotics	Formulation-specific RCTs for E. coli Nissle 1917 and a multistrain product [49,50]	Efficacy is formulation-specific, and guideline-supported use remains limited to clinical-trial settings [78]	Verify product identity, viability, dose, persistence, and background therapy
Synbiotics	One small B. longum/inulin-FOS pilot [73]	Mechanistic and histologic signals require confirmation in adequately powered trials	Use factorial designs to separate organism, substrate, and combination effects
Postbiotics	No qualifying UC RCT was identified under the consensus definition [77]	Evidence for purified metabolites is considered separately	Define source, inactivation, composition, batch consistency, and target engagement
SCFA/butyrate	Controlled rectal trials were negative overall or inconsistent, while a small oral sodium butyrate trial reported biomarker and patient-reported signals [42,43,74]	Findings vary by route, endpoint, and study; routine efficacy remains uncertain	Stratify location, delivery, epithelial competence, and baseline fermentation
FMT	Positive and negative RCTs [51,52,53,75]	Randomized proof of concept with substantial protocol dependence; conventional FMT remains trial-restricted [54]	Standardize donor screening, preparation, dose, comparator, safety, and durability
VNS	The principal adult pilot involved Crohn’s disease; randomized UC efficacy data are unavailable [47]	Investigational for anti-inflammatory and symptom-directed indications	Sham control, target engagement, objective mucosal outcomes, and device safety
5-HT/neuropeptide targets	Human tissue and symptom associations, with causal data mainly preclinical [25,31,32]	Receptor- and phenotype-specific investigation	Separate symptom control from disease modification

Table note: Interpretation is formulation- and indication-specific. Most approaches remain adjunctive or investigational alongside guideline-based UC therapy. Abbreviations: 5-HT, 5-hydroxytryptamine; FMT, fecal microbiota transplantation; FOS, fructo-oligosaccharide; RCT, randomized controlled trial; SCFA, short-chain fatty acid; UC, ulcerative colitis; VNS, vagus nerve stimulation.

## Data Availability

The original contributions presented in the study are included in the article/Supplementary Material, further inquiries can be directed to the corresponding authors.

## Supplementary Materials

The following supporting information can be downloaded at https://www.mdpi.com/article/10.3390/biomedicines14102361/s1, Supplementary File S1: Reproducible PubMed Search Strategy: search date and coverage, complete field-tagged queries, query-level yields, PMID-based deduplication, eligibility and prioritization criteria, evidence labels, and the module-based selection process; Supplementary File S2: Selected Pivotal Evidence Matrix: design, evidence level, population or model, supported interpretation, primary limitation, verified citation, and manuscript location for 30 pivotal studies [6,7,8,9,13,14,15,21,22,25,29,31,39,42,43,47,48,49,50,51,52,53,57,66,67,68,69,73,74,75].
