# Supplementary material for: Gut Microbiota-Driven Neuroimmune Crosstalk in Ulcerative Colitis: From Mechanisms to Therapeutic Implications
Source: Biomedicines. 2026 Oct 16;14(10):2361. doi: 10.3390/biomedicines14102361 (PMC13702003; doi:10.3390/biomedicines14102361)
Supplement: Supplementary file 1 [file biomedicines-14-02361-s001.zip › Supplementary File S1.pdf]

Supplementary File S1. Reproducible PubMed Search Strategy

Review type: Mechanism-focused structured narrative review. Database: PubMed. Search date: 26 September 2026. Date coverage: 1 January 1800 to 26 September 2026.

The eight queries returned 7,528 records before deduplication and 6,748 unique PMIDs. This output was used as a broad citation-discovery pool, not as a PRISMA systematic-review corpus. No dual independent screening, registered protocol, or duplicate systematic data extraction is claimed.

Query	Records	Exact PubMed expression
Q1_microbiota_neuroimmune	120	(("Colitis, Ulcerative"[Mesh] OR "Inflammatory Bowel Diseases"[Mesh] OR "ulcerative colitis"[Title/Abstract] OR "inflammatory bowel disease"[Title/Abstract] OR IBD[Title/Abstract]) AND (microbiota[Title/Abstract] OR microbiome[Title/Abstract]) AND (neuroimmune[Title/Abstract] OR "enteric nervous system"[Title/Abstract] OR "vagus nerve"[Title/Abstract] OR "enteric glia"[Title/Abstract])) AND ("1800/01/01"[Date - Publication] : "2026/09/26"[Date - Publication])
Q2_SCFA	563	(("Colitis, Ulcerative"[Mesh] OR "Inflammatory Bowel Diseases"[Mesh] OR "ulcerative colitis"[Title/Abstract] OR "inflammatory bowel disease"[Title/Abstract] OR IBD[Title/Abstract]) AND ("short-chain fatty acids"[Title/Abstract] OR butyrate[Title/Abstract] OR SCFA[Title/Abstract]) AND (immune[Title/Abstract] OR neuron*[Title/Abstract] OR vagus[Title/Abstract] OR enteric[Title/Abstract])) AND ("1800/01/01"[Date - Publication] : "2026/09/26"[Date - Publication])
Q3_tryptophan_indole_AhR_serotonin	486	(("Colitis, Ulcerative"[Mesh] OR "Inflammatory Bowel Diseases"[Mesh] OR "ulcerative colitis"[Title/Abstract] OR "inflammatory bowel disease"[Title/Abstract] OR IBD[Title/Abstract]) AND (tryptophan[Title/Abstract] OR indole*[Title/Abstract] OR AhR[Title/Abstract] OR serotonin[Title/Abstract] OR 5-HT[Title/Abstract]) AND (microbiota[Title/Abstract] OR microbiome[Title/Abstract])) AND ("1800/01/01"[Date - Publication] : "2026/09/26"[Date - Publication])
Q4_bile_acid_FXR_TGR5	574	(("Colitis, Ulcerative"[Mesh] OR "Inflammatory Bowel Diseases"[Mesh] OR "ulcerative colitis"[Title/Abstract] OR "inflammatory bowel disease"[Title/Abstract] OR IBD[Title/Abstract]) AND ("bile acid"[Title/Abstract] OR "bile acids"[Title/Abstract] OR FXR[Title/Abstract] OR TGR5[Title/Abstract]) AND (microbiota[Title/Abstract] OR immune[Title/Abstract] OR neuron*[Title/Abstract])) AND ("1800/01/01"[Date - Publication] : "2026/09/26"[Date - Publication])
Q5_vagus_cholinergic	180	(("Colitis, Ulcerative"[Mesh] OR "Inflammatory Bowel Diseases"[Mesh] OR "ulcerative colitis"[Title/Abstract] OR "inflammatory bowel disease"[Title/Abstract] OR IBD[Title/Abstract]) AND ("vagus nerve"[Title/Abstract] OR "vagus nerve stimulation"[Title/Abstract] OR "cholinergic anti-inflammatory pathway"[Title/Abstract] OR "alpha7 nicotinic"[Title/Abstract])) AND ("1800/01/01"[Date - Publication] : "2026/09/26"[Date - Publication])
Q6_enteric_glia	120	(("Colitis, Ulcerative"[Mesh] OR "Inflammatory Bowel Diseases"[Mesh] OR "ulcerative colitis"[Title/Abstract] OR "inflammatory bowel disease"[Title/Abstract] OR IBD[Title/Abstract]) AND ("enteric glia"[Title/Abstract] OR "enteric glial cells"[Title/Abstract] OR "glial fibrillary acidic protein"[Title/Abstract])) AND ("1800/01/01"[Date - Publication] : "2026/09/26"[Date - Publication])
Q7_sensory_neuropeptide	457	(("Colitis, Ulcerative"[Mesh] OR "Inflammatory Bowel

		Diseases"[Mesh] OR "ulcerative colitis"[Title/Abstract] OR "inflammatory bowel disease"[Title/Abstract] OR IBD[Title/Abstract]) AND ("sensory neuron"[Title/Abstract] OR CGRP[Title/Abstract] OR "substance P"[Title/Abstract] OR VIP[Title/Abstract] OR neuropeptide[Title/Abstract])) AND ("1800/01/01"[Date - Publication] : "2026/09/26"[Date - Publication])
Q8_microbiota_neuromodulation_therapy	5028	(("Colitis, Ulcerative"[Mesh] OR "Inflammatory Bowel Diseases"[Mesh] OR "ulcerative colitis"[Title/Abstract] OR "inflammatory bowel disease"[Title/Abstract] OR IBD[Title/Abstract]) AND (probiotic*[Title/Abstract] OR prebiotic*[Title/Abstract] OR postbiotic*[Title/Abstract] OR synbiotic*[Title/Abstract] OR "fecal microbiota transplantation"[Title/Abstract] OR "faecal microbiota transplantation"[Title/Abstract] OR FMT[Title/Abstract] OR "vagus nerve stimulation"[Title/Abstract])) AND ("1800/01/01"[Date - Publication] : "2026/09/26"[Date - Publication])

Eligibility and prioritization

- Prioritized: human UC interventions, longitudinal cohorts, mucosal tissue studies, and studies connecting biological measurements to disease activity or symptoms.
- Used with explicit qualification: mixed-IBD human studies when UC-specific results were unavailable; experimental colitis, gnotobiotic, ex vivo, and in vitro studies for causal or cellular mechanisms.
- De-emphasized: descriptive taxonomy without functional interpretation; general gut-brain studies without mucosal immune relevance; psychiatric-comorbidity studies without mechanistic data; Crohn's disease-only therapeutic findings presented as UC evidence.
- Evidence labels: A, direct human UC intervention; B, human UC observational/tissue; C, mixed human IBD; D, in vivo experimental; E, ex vivo/in vitro or foundational non-UC mechanism; H, author-derived hypothesis.

From the discovery pool to the final citation set

Selection was iterative and module-based. Candidate records were considered within the eight search domains and retained only when they supported a specific statement, defined a clinical or terminological boundary, supplied a necessary experimental mechanism, or documented an evidence gap. Original studies were preferred over reviews for factual claims. Because this was not a formal sequential screening process, intermediate title/abstract and full-text exclusion counts were not prospectively logged and were not retrospectively estimated.

Stage	Count	Description
Query output	7,528	Sum of records returned by the eight predefined PubMed queries.
Unique discovery pool	6,748	Unique PMIDs after deduplication; used for citation discovery, not a PRISMA screening denominator.
Module-based consideration	Not prospectively counted	Iterative assessment by relevance, evidence level, claim support, and availability of original research.
Final cited set	78	References retained because they directly

		support a statement, boundary, mechanism, intervention, or evidence gap in the manuscript.
Selected pivotal-evidence matrix	30	Subset mapped in detail for design, evidence level, interpretation, limitation, reference number, and manuscript location.
